# *Critical Alliance* of AI in Education: A Pedagogical Framework for Safeguarding Cognitive Skills

**DOI:** 10.3390/ime5010022

**Published:** 2026-02-04

**Authors:** Marcos J. Ramos-Benitez, Martha E. García-Osorio, Yamixa Delgado

**Affiliations:** 1 Department of Basic Science, Microbiology Division, Ponce Health Sciences University, Ponce, PR 00716, USA; 2 School of Medicine, Universidad Central del Caribe, Bayamón, PR 00960, USA; 3 School of Chiropractic, Universidad Central del Caribe, Bayamón, PR 00960, USA; 4 Department of Biochemistry and Pharmacology, San Juan Bautista School of Medicine, Caguas, PR 00725, USA

**Keywords:** artificial intelligence, critical alliance, education, critical thinking, large language models

## Abstract

The integration of artificial intelligence (AI), particularly large language models (LLMs), into education, marks a profound shift in how knowledge is accessed, processed, and applied. These tools offer clear advantages—including improved efficiency, immediate support, and high productivity—but it may simultaneously weaken foundational skills. This Perspective examines the dual impact of AI on education, arguing that over-reliance on AI may displace essential cognitive processes that reinforce professional competence. Emerging evidence points to troubling associations between frequent AI use and diminished critical reasoning. We propose a model of *critical alliance*, in which AI augments but does not replace core intellectual processes. Unlike existing AI competency or digital literacy, this model centers on preserving human cognitive agency, judgment, reflection, and intellectual ownership, as primary educational outcomes. This framework not only emphasizes cognitive independence, but also equitable access, ethical vigilance, and faculty development as cornerstones of AI literacy. Addressing these questions is essential to safeguard both intellectual growth and educational equity in an AI-augmented era. Unlike existing digital literacy or AI competency frameworks, the *critical alliance* explicitly centers on preserving human cognitive agency and intellectual ownership as educational priorities, particularly in environments increasingly shaped by high-performing generative systems.

## 1. AI Inflection Point in Education

We are living through a period of profound technological transformation, marked by the rapid integration of artificial intelligence (AI) into nearly every facet of human activity. Generative large language models (LLMs)—for example, ChatGPT, Copilot, and Gemini—capable of producing human-like text, engaging in complex dialogue, and processing information at scale, have moved from specialized research labs into mainstream use with great speed. Higher education, including medical and scientific training, is experiencing this shift acutely, as these tools saturate learning environments and professional workflows. The pace of this integration often outstrips our capacity for deliberate adaptation and critical reflection on its long-term consequences.

While powerful AI tools offer undeniable opportunities for education, they simultaneously present deep challenges. Supporters highlight AI’s potential to enhance learning efficiency, offer personalized academic support, automate routine tasks for educators, and introduce innovative teaching methodologies [1–3]. These capabilities promise smoother, more engaging, and potentially more effective educational experiences. However, alongside this potential lies a growing concern: the impact of AI use on the development and maintenance of fundamental cognitive skills, particularly critical thinking, reflection, synthesis and independent reasoning [3–7]. Individuals educated before the AI era acquired knowledge through effortful searching, comparison, and iteration—practices that built cognitive resilience and analytical depth. As AI systems begin to intermediate many of these tasks, the potential erosion of such skills must be scrutinized.

This paper introduces the concept of a *critical alliance*, a pedagogical framework designed to navigate this dual landscape of opportunity and risk. Unlike conventional AI literacy models that emphasize technical proficiency or frameworks grounded solely in metacognitive coaching, the *critical alliance* reframes AI not just as a tool to be mastered, but as a cognitive mediator whose use demands ethical, epistemic, and social evaluation. The framework’s central innovation lies in prioritizing human cognitive agency, intellectual ownership, and ethical discernment as non-negotiable outcomes in an AI-augmented educational landscape.

Rather than adapting human cognition to the logic of automated systems, the *critical alliance* insists on preserving learner autonomy through intentional, reflective, and critically engaged interactions with AI. It positions educators not merely as technical guides, but as stewards of human intelligence, tasked with fostering the judgment, humility, and discernment needed to engage AI as a partner, never a substitute, in the process of learning.

## 2. AI’s Utility and Cognitive Risk

The integration of AI into education presents a paradox: it offers significant gains in efficiency and personalization while simultaneously posing risks to core cognitive functions. Understanding both sides of this equation is essential for responsible adoption. We are not romanticizing the past or rejecting technology. As faculty members in health professions and medical education, we see students using AI to study, summarize, and brainstorm, often with enhanced ease. However, challenges emerge when learners are asked to interpret scientific data, critique the literature, or generate hypotheses. These are the moments when the absence of well-developed critical thinking becomes evident. Several studies have suggested potential negative associations between frequent AI use and critical thinking abilities in young learners [3–7].

We define cognitive independence as the learner’s capacity to synthesize, verify, and apply information without undue reliance on automated systems. This definition aligns with AI competency frameworks proposed by Russell et al. [8] and the Macy Foundation innovation reports [9,10], which highlight independent reasoning as essential in AI-augmented learning environments. To better understand the nature of this cognitive risk, recent studies offer key insights.

For students, LLMs provide immediate access to extensive information, streamlining retrieval, and aiding in the summarization of complex texts. They can assist in academic writing across multiple stages, from generating arguments and drafting content to refining grammar and style [11]. One widely cited argument in favor of AI is that it may help “free up” cognitive resources. By automating low-level tasks like information gathering and summarization, students might be able to dedicate more mental energy to critical analysis, creative reasoning, and deeper conceptual integration [12]. Yet this promise is not without caveats. If students routinely offload essential learning tasks to AI, they may bypass the very cognitive struggles that foster critical thinking. Core academic activities, like comparing sources, evaluating claims, and synthesizing ideas, are effortful but necessary for intellectual growth [12]. When AI is used to bypass key cognitive processes, overreliance on such tools may weaken higher-order thinking skills.

A deeper concern lies in the delegation of core cognitive functions, such as memory recall, problem-solving, and analytical reasoning, to AI systems. Empirical studies are beginning to show significant negative associations between frequent AI use and students’ critical thinking performance, with cognitive offloading acting as a key mediating factor [4,7,8]. Several studies cited in this section used mixed methods to assess the relationship between AI use and cognitive performance. For example, recent evidence from secondary education showed that access to GPT-4–based math tutors substantially boosted students’ immediate performance but, when removed, led those to learn less and perform worse than peers without AI [13]. In higher education, Zhai et al. (2024) conducted a systematic review of 27 studies, identifying a consistent pattern where over-reliance on LLMs was associated with reduced engagement in deep learning strategies [3]. Vieriu and Petrea’s study (2025) used survey-based metrics from over 500 university students and found statistically significant negative correlations between daily AI use and performance on validated critical thinking tasks [7]. Additionally, Kosmyna et al. (2025) employed EEG data to measure brain connectivity and cognitive load in 54 participants completing writing tasks with and without AI [14]. The group using AI tools showed markedly lower neural activation in prefrontal and parietal regions, supporting concerns of reduced cognitive effort. While more longitudinal data is needed, these findings collectively strengthen the claim that habitual AI use may hinder the development of critical reasoning skills in educational settings.

It is important to acknowledge that the long-term cognitive impacts of AI are not yet fully understood. Most of the studies cited in this article are cross-sectional and demonstrate correlational links rather than definitive causal relationships. While the evidence base remains limited and context-dependent, recent longitudinal and experimental studies offer early insights into how sustained AI use may shape cognitive skills over time. Experimental findings further highlight potential risks associated with overuse. For example, a recent four-week randomized study found that heavy users of ChatGPT, particularly in its voice modality, exhibited increased emotional dependence and reduced social engagement [15]. Although conducted outside of formal educational settings, these findings illustrate how sustained reliance on AI tools can displace essential human functions, paralleling concerns about cognitive offloading in learning contexts. Current and future longitudinal studies will be critical to building a more complete picture of these dynamics, helping to disentangle correlation from causation and to identify the conditions under which AI use supports—not undermines—intellectual growth and professional competence.

While our examples emphasize medical and scientific education, the concerns we raise apply broadly across all levels of education. The cognitive risks and potential challenges to authorship associated with uncritical AI use—such as diminished memory encoding, shallow reasoning, and erosion of authorial contribution—are equally relevant in K–12 settings and in informal learning environments. Initiatives such as the metaLAB, Harvard, Berlin & Basel and AI Pedagogy Project are beginning to address these issues through curricular design and educator training that foster responsible AI engagement among diverse learner populations [16,17]. Additionally, Microsoft’s report on “Bridging the AI Literacy Gap” highlights the need to develop critical thinking and AI literacy across all sectors of society, not just within professional training [18]. Expanding access to pedagogical tools that cultivate cognitive independence is therefore essential to fulfilling education’s broader societal mission.

Ultimately, persistent over-reliance on AI risks weakening foundational domains such as analysis, reasoning, and communication [12–14,19]. This is especially concerning in professional training programs, where learners must become not just efficient tool users, but also competent, independent thinkers. Without intentional pedagogical safeguards, we risk producing graduates who lack the cognitive agility and judgment required for responsible practice in complex, high-stakes environments [9,10]. Also, we should recognize that the cognitive impact of AI is shaped both by individual differences and by structural inequities. At the learner level, factors such as prior knowledge, metacognitive skills, and motivation influence how students engage with AI tools. Some may critically evaluate outputs to deepen understanding, while others may become over-reliant and disengaged, especially those lacking the cognitive scaffolding or self-regulation strategies to assess AI-generated content [14]. At the same time, access to AI is not evenly distributed. Emerging evidence points to an ‘AI literacy divide,’ where students from under-resourced regions often have limited exposure to AI-assisted learning and reduced technological capital [20]. This divide is exacerbated in minority and first-generation students, who show lower engagement and less ability to apply AI knowledge beyond coursework compared to their peers [21]. In contrast, students with frequent access may gain fluency but also face heightened risks of over-reliance. Thus, the call for a *critical alliance* with AI is not a one-size-fits-all condemnation but a pedagogical imperative: to foster wise and intentional use among all learners, while addressing disparities through equitable access and inclusive AI literacy initiatives.

**Figure 1.**
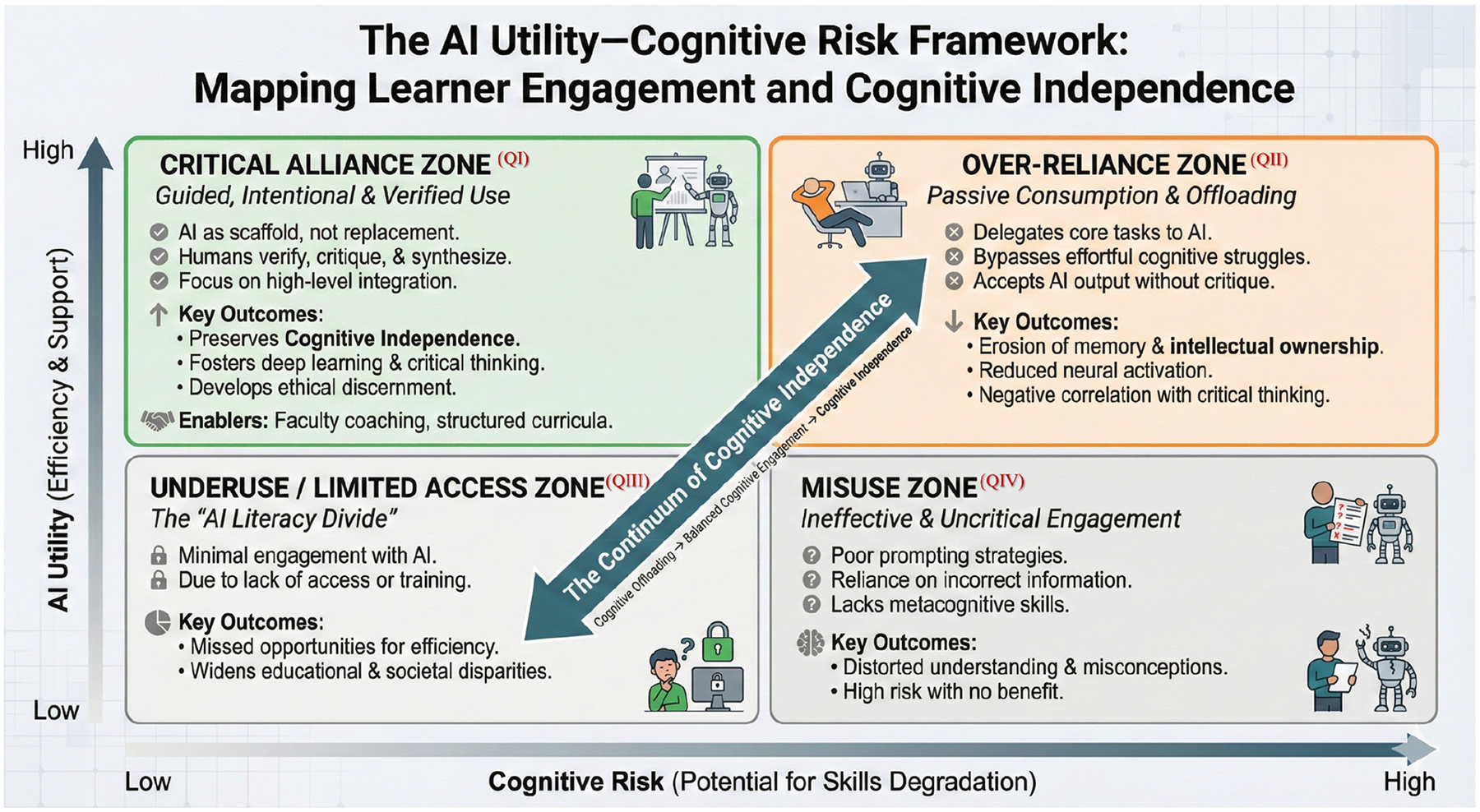
The AI Utility–Cognitive Risk Framework: Mapping Learner Engagement and Cognitive Independence. *Critical Alliance* Zone (QI) promotes critical thinking and deep learning. Over-Reliance Zone (QII) reduces critical thinking. Underuse/Limited Access Zone (QIII) shows minimal use. Misuse Zone (QIV) promotes reliance on incorrect information. The central diagonal double-headed arrow labeled “The Continuum of Cognitive Independence” represents the conceptual spectrum from cognitive offloading to balanced cognitive engagement to cognitive independence. The arrow is not prescriptive; it does not indicate a recommended direction. The pedagogical goal is to maintain learners in the *Critical Alliance* Zone, avoiding shifts toward extremes.

To contextualize these findings in this section, Figure 1 presents a schematic framework in the form of a quadrant model, which is divided into four sections (Q I–IV), that highlights how AI’s benefits and risks interact to shape critical thinking and cognitive development. Students’ engagement with AI can be mapped along a continuum where increasing utility is accompanied by rising cognitive risk when cognitive independence is not maintained. The purpose of the diagram is to show that a learner starting in any sub-optimal zone (QII: Over-Reliance, QIII: Underuse, or QIV: Misuse) must undergo a cognitive transformation toward *Critical Alliance* Zone (QI). Students, even when properly trained, can easily slip back into QII, especially under pressure or when performing routine tasks. The central arrow is double headed to emphasize that QI is a dynamic state, not a static destination. It is placed to show the gradient of risk and how states are distributed along this continuum, making it more conceptual than directional. This suggests that maintaining balanced cognitive engagement requires continuous effort, metacognitive self-regulation, and pedagogical scaffolding. Educators must constantly work to keep the learner in QI and pull them away from the offloading states.

## 3. Imperative of Human Judgment

The AI’s remarkable ability to produce fluent and coherent information poses a significant challenge for users, particularly those with limited expertise, in discerning reliable information from believable falsehoods. Treating eloquence as proof of truth is a major weakness in the AI era.

Considering this emerging evidence, we propose a foundational principle for navigating AI in educational settings, particularly in health professions training: if an individual lacks the requisite knowledge and critical skills to independently evaluate the accuracy, relevance, and potential biases of an AI-generated output, they should refrain from relying on it for substantive decisions. In domains like science, health care, and policy, where the errors can have serious consequences, rigorous verification, epistemic humility, and critical thinking are not negotiable but indispensable prerequisites for the responsible use of AI.

Beyond technical skills, educators must also prepare learners to recognize and mitigate specific ethical challenges introduced by AI. These include automation bias, where users over-trust AI outputs regardless of accuracy, and hallucinations, where AI systems produce plausible but false or misleading information [22]. Without structured ethical reasoning training, students may not possess the tools to critically evaluate AI-generated content. As highlighted by recent faculty development models such as the CRAFT (Center for Research on Foundation Models, Stanford University) framework at Stanford [23] and milestone-based guides by Khamis et al., [24] education must evolve to include epistemic vigilance, fairness, transparency, and accountability in AI use. These frameworks promote an educator’s role as a coach of ethical discernment and cognitive stewardship, crucial for cultivating judgment in AI-enhanced environments. Reliance on AI without the cognitive and ethical capacity for evaluation may constitute a resignation of intellectual and professional responsibility. This principle underpins the faculty development strategies discussed in Sections 4 and 5, where models such as CRAFT framework and curriculum-level approaches operationalize the educator’s role as an ethical and cognitive coach, linking conceptual guidance to practical implementation through curriculum design, pilot programs, and evaluation frameworks.

## 4. Re-Evaluation of Pedagogical Practices

These findings reinforce our call for a *critical alliance*, a pedagogical model that integrates AI while deliberately preserving learners’ cognitive independence, ethical discernment, and reflective capacities. Unlike traditional metacognitive or AI literacy frameworks that focus primarily on individual skill development or tool proficiency, the *critical alliance* reframes AI as a cognitive mediator whose use sustained pedagogical scaffolding and human judgement. Integrating AI demands a fundamental shift in what and how we teach. Since AI can effortlessly generate information and handle routine tasks, education must move beyond memorization and basic understanding to foster advanced cognitive capacities. These unique human abilities: critical analysis, evidence evaluation, logical reasoning, creative problem-solving, ethical judgment, and adaptability, are vital for navigating our increasingly complex, AI-integrated education learning environments.

Our goal is not just to teach students how to use AI; it is to empower them to think critically with, through, and about these powerful tools. Achieving this transformation requires specific teaching strategies that promote critical thinking, epistemic vigilance, and ethical discernment in the context of AI. Rather than viewing AI as a source of ready-made answers, educators can leverage it to deepen inquiry, provoke discussion, and engage students in authentic intellectual struggles. Importantly, the *critical alliance* does not assume expert-level AI knowledge; instead, it emphasizes scaffolded development of judgment, supported by guided practice and collaborative sense-making.

At the same time, the framework explicitly acknowledges the epistemic asymmetry between human learners and large-scale AI systems. Contemporary LLMs operate at a scale, speed, and breadth of representation that no individual learner or educator can fully comprehend. The *critical alliance* therefore does not require users to understand AI systems at a level comparable to their generative complexity. Instead, it promotes bounded understanding and calibrated trust, equipping learners with strategies such as triangulation, source verification, plausibility checking, and bias detection, to critically evaluate AI outputs despite incomplete technical transparency. In this sense, the alliance responds to AI’s scale not by demanding technical mastery, but by strengthening human judgment, contextual reasoning, and ethical oversight in the face of asymmetry.

This *critical alliance* is operationalized through intentional instructional strategies that promote guided engagement with AI, rather than passive dependence. For example, students may be asked to compare their own diagnostic reasoning or writing output to an AI-generated version and explicitly critique its logic, evidence of use, and bias. Faculty act not as content dispensers, but as reflective facilitators who coach students to question, verify, and improve AI responses. This model fosters epistemic humility, analytical depth, and ethical reasoning while encouraging the productive use of AI as a tool. Projects such as Stanford’s CRAFT framework and the AI Pedagogy Project exemplify this approach, offering modular exercises where students interrogate AI performance, reflect on its impact, and build their own intellectual agency.

The *critical alliance* model is grounded in established learning theory while extending it to address AI-specific dynamics. It aligns with constructivist learning theory, which emphasizes active knowledge construction [25]. By encouraging learners to critique AI-generated outputs, the model leverages Vygotsky’s concept of the ‘zone of proximal development’, positioning AI as a scaffold rather than a substitute for cognitive effort. From the perspective of cognitive load theory, the alliance strategically offloads low-level tasks to preserve students’ capacity for deep analysis and reasoning [26]. Furthermore, it aligns with connectivism by framing AI as a powerful node within modern learning networks that must be engaged with and managed critically [27].

This framework further aligns with human-in-the-loop systems, in which preserving human oversight and judgment is essential for reliability and ethical deployment [28]. Because AI systems lack intention, responsibility, and social accountability, meaning-making and ethical judgment must remain firmly situated in humans. Importantly, AI-generated outputs should not be treated as neutral or authoritative knowledge artifacts. LLMs generate text without intention, responsibility, or accountability; they do not participate in social meaning-making, nor can they be held epistemically or ethically responsible for their outputs. The cognitive and ethical burden therefore lies entirely with human users, who must interpret, contextualize, and evaluate AI-generated information within their disciplinary, social, and professional communities. The *critical alliance* framework explicitly centers this asymmetry: AI produces signals, but humans remain accountable meaning-makers. Education, in this view, is not about trusting AI outputs, but about cultivating learners’ capacity to interrogate, situate, and responsibly appropriate them in socially consequential contexts.

To operationalize the concept of a *critical alliance*, we advocate for a shift in the educator’s role from content transmitter to ethical and cognitive coach. This aligns with pedagogical frameworks such as Corbeil et al.’s curriculum-level approach for teaching critical thinking for ethical AI engagement [29]. Educators can implement strategies such as AI critique exercises, structured reflections on generative outputs, and role-play scenarios in which students identify misinformation, recognize automation bias, or challenge hallucinated claims. Traditional instruction remains valuable in establishing foundational knowledge, but within the *critical alliance* it functions as a springboard for reflective, dialogic, and evaluative engagement with AI.

Faculty can further model critical AI engagement by transparently demonstrating how they verify AI-assisted outputs, articulate ethical dilemmas, and weigh trade-offs in AI-supported reasoning. These instructional moves promote a reflective classroom culture, fostering humility, and collaborative inquiry. Institutions may support this transition through milestone-based faculty development programs that scaffold progression from basic AI literacy to coaching roles, as outlined by Khamis et al. [24]. Recognizing that not all learners or educators begin with advanced AI competence, the alliance model emphasizes scaffolded development, progressing from foundational AI literacy to deeper ethical discernment and cognitive stewardship.

Platforms such as the AI Pedagogy Project [17] offer actionable teaching resources, such as curated prompts, activities, and implementation guides, designed to help educators integrate AI while strengthening student autonomy, ethical reflection, and civic responsibility. To support this transition, we propose several innovative considerations for educators, curriculum developers, and policymakers (Table 1). These include integrating AI literacy into core curricula, embedding reflection checkpoints into AI-assisted learning, and using dynamic rubrics to evaluate not only academic performance but also the nature of students’ engagement with AI. Successfully making this pedagogical change will not be easy. It calls for a significant evolution in how educators think, the skills they possess, and the institutional structures that support them.

## 5. Implications for Educators, Institutions and Key Questions

The accelerating integration of AI into medical, scientific, and broader educational contexts presents both a powerful tool and a profound pedagogical test. While these technologies offer efficiency, personalization, and innovation, their uncontrolled application could diminish essential cognitive, ethical, and reflective capacities, those very qualities that define competent and responsible professionals and citizens. Educators must urgently rethink their role, not just teaching students to use AI, but to think critically with it. This includes fostering cognitive independence, addressing automation bias, and encouraging ethical reasoning. These should not be addressed as optional enhancements; they are foundational elements of future-ready education.

Institutional readiness is equally critical. Curricula must evolve to embed AI within pedagogies that promote reflection, inquiry, and conscious vigilance. Faculty development should be structured around milestone-based frameworks to support progression from technical competence to ethical stewardship. Assessment systems must incorporate tools that gauge not only performance, but also the nature of learners’ AI engagement and reasoning strategies.

In the short term, our efforts should focus on translating the *critical alliance* framework into applied educational practice through pilot implementations and case-based demonstrations. These initiatives could embed guided AI–human interaction modules within existing curricula, particularly in medical and scientific education, to evaluate their effectiveness in strengthening cognitive independence, critical reasoning, and ethical discernment. Pilot programs might include AI-contrast learning exercises, where students critique and refine AI-generated outputs, and faculty-led reflective assessments that monitor shifts in cognitive load, judgment, and engagement. Complementary to this conceptual approach, Ahmed et al. (2023) provides practical examples of how ChatGPT can be integrated into medical curricula to enhance learning, feedback, and assessment processes, offering the applied “how” and “what” that parallel this framework’s theoretical “why” and “how to be careful” [30].

Evaluation of these pilots should combine quantitative measures (e.g., validated critical-thinking rubrics, metacognitive self-assessments, learning analytics) with qualitative analyses (e.g., reflective journals, faculty observations, focus groups) to capture both cognitive and affective dimensions of learning. By systematically documenting outcomes, such case-based applications will provide empirical grounding for the *critical alliance* framework, demonstrating its scalability, adaptability, and impact across diverse disciplines and institutional contexts. Ultimately, operationalizing the *critical alliance* through iterative pilot studies will allow educators to refine the model in real-world settings, transforming it from a conceptual perspective into a tested pedagogical strategy that safeguards human cognition while responsibly integrating AI into education. Faculty development, assessment strategies, and institutional policies must evolve to ensure that technology remains a means, not a substitute, for learning.

On the other hand, the comprehensive review on the use of AI in clinical supervision by Abdulnour et al. (2025) highlights that the widespread use of AI among young learners creates an inversion of expertise, where students may often possess greater technological fluency than their instructors [6]. To address this challenge, the authors argue that professional development in science and health education must be redesigned to include AI literacy and shared learning models. These models should encourage educators, students, and other stakeholders to learn together in reflective and collaborative ways, reframing uncertainty around AI into an opportunity to strengthen critical thinking across the entire educational community.

Key questions remain open for discussion:

- How can we rigorously evaluate the cognitive impact of AI use across different stages of learning, particularly among learners with diverse backgrounds, motivation levels, and metacognitive strengths?
- Which teaching models best support cognitive independence, ethical discernment, and resilience in the face of misinformation and over-reliance on AI tools?
- How can institutions scale faculty development and curricular reform in ways that preserve human judgment and promote equitable, responsible AI integration?
- What safeguards are needed to ensure AI does not exacerbate educational inequities, particularly for learners from underrepresented or underserved backgrounds who may be disproportionately affected by over-reliance on AI for feedback, guidance, or instruction?

## 6. Conclusions

By thoughtfully navigating the integration of AI in education, we can preserve the intellectual and ethical dimensions that define meaningful learning. Our proposed *critical alliance* invites institutions and educators to foster cognitive independence, reflective practice, and ethical reasoning as foundational competencies in an AI-augmented world. Also, a central challenge for education is not only to foster critical use, but also to ensure equitable access. By embracing the *critical alliance*, education can navigate these challenges, empowering learners to harness technology not as a replacement for the mind, but as a catalyst for a more thoughtful, rigorous, and ultimately more human intelligence.

## Figures and Tables

**Table 1.** Strategies to foster critical thinking and keep the learner in the *Critical Alliance* Zone in AI-integrated education.

Idea	Description	Application for Educators
AI literacy as core competency	Position AI literacy (not just technical use, but understanding its limits, biases, and cognitive impacts) as a required competency in medical and scientific education.	Incorporate AI ethics, critical evaluation of AI outputs, and cognitive impacts of AI into core curricula and accreditation standards.
Critical thinking through AI contrast	Use AI-generated answers as a foil for deeper engagement: ask students to critique, verify, or improve AI outputs.	Develop assignments where students must detect errors, biases, or logical flaws in AI responses, strengthening critical appraisal skills.
Cognitive load monitoring policies	Encourage policies that monitor and balance cognitive load in AI-enhanced learning environments to prevent over-reliance.	Implement reflective checkpoints: after AI use, students must document what tasks they automated versus what they reasoned through manually.
Dynamic AI use rubrics	Create flexible evaluation rubrics that reward critical use of AI, penalizing passive acceptance of AI-generated material.	In grading, assess how students use AI: Did they independently verify? Did they expand upon or merely repeat AI outputs?
Human intelligence preservation benchmarks	Propose national or institutional benchmarks that measure cognitive independence and critical reasoning alongside AI use proficiency.	Develop standardized evaluations to ensure that human cognitive skills are preserved and strengthened, not eroded, through AI-integrated education.
